# Evaluation of an Immunoassay-Based Algorithm for Screening and Identification of *Giardia* and *Cryptosporidium* Antigens in Human Faecal Specimens from Saudi Arabia

**DOI:** 10.1155/2014/213745

**Published:** 2014-01-29

**Authors:** Yousry Hawash

**Affiliations:** ^1^Department of Medical Parasitology, National Liver Institute, Menoufia University, Shebin-El Koum 32513, Menoufia, Egypt; ^2^Department of Medical Laboratory Science, College of Applied Medical Science, Taif University, Taif 2425, Saudi Arabia

## Abstract

An immunoassay-based algorithm, involving three commercial kits, was introduced and evaluated for screening and identification of *Giardia/Cryptosporidium* antigens in human stool specimens. Initially, *Giardia/Cryptosporidium* Chek kit (TechLab), an enzyme-linked immunosorbent assay (ELISA), was adopted for screening. The ELISA-positive reactions were subsequently characterised by RIDA Quick *Giardia* and RIDA Quick *Cryptosporidium* immunochromatographic kits (R-Biopharm). A gold standard test comprising PCR and microscopy was used for preparing control samples. Performance of individual kits was tested against these samples which included 50 *Giardia*-positive, 40 *Cryptosporidium*-positive, and 70 *Cryptosporidium/Giardia*-negative. For *Cryptosporidium*, specificities of the ELISA and RIDA Quick *Cryptosporidium* kits were 95.71% and 100%, respectively. Both kits demonstrated sensitivity of 95%. For *Giardia*, the ELISA and RIDA Quick *Giardia* kits showed sensitivities of 100% and 97.5%, respectively. Specificities obtained by the ELISA and RIDA Quick *Giardia* were 95.7% and 100%, respectively. Based on the results of two reference PCRs, on 250 random samples, the algorithm exhibited sensitivity, specificity, positive predictive value, and negative predictive value of 97.06%, 100.00%, 100.00%, and 98.91%, respectively. In conclusion, this immunoassay-based algorithm can be used as routine test in diagnostic laboratories for screening and identification of a large number of samples.

## 1. Introduction

*Cryptosporidium* and *Giardia *are two common intestinal protozoa causing gastroenteritis in humans [1, 2]. Many outbreaks of diarrhoea have been frequently attributed to these two protozoa [3]. Infections are often common in children and patients with weakened immunity [4, 5]. Definitive diagnosis of these protozoa requires the identification of their morphological stages, trophozoite or cyst stages of *Giardia, *and oocyst stage of *Cryptosporidium* in faecal specimens. In developing countries, this is routinely done through classical microscopic-based techniques, namely, the iodine-saline mount for *Giardia *and the modified Ziehl-Neelsen (mZN) staining for *Cryptosporidium* [6, 7]. However, these methods lack sensitivity due to the intermittent shedding or the presence of organisms in low numbers [8]. Accordingly, antigen detection immunoassays have been developed and adopted in many hospitals and public health laboratories for the diagnosis of these two protozoa [9]. The direct fluorescent-antibody tests (DFA) detecting intact organisms and the enzyme immunoassays (EIAs) detecting protozoan soluble antigens in stool are the two widely used antigen detection immunoassays [9, 10]. The sensitivity and specificity of DFA have been reported to be 96 to 100% and 99 to 100%, respectively [9–13]. In the other hand, the sensitivity and the specificity of EIAs have been reported to be 94 to 97% and 99 to 100%, respectively [9, 11, 13]. However, a large number of faecal specimens can be screened at one time with EIAs with less technical skills, less costs, less labour, and less laboratory turnaround times [14, 15]. In addition, rapid immunochromatographic-based kits have been developed and became widely used for detection of *Cryptosporidium* and *Giardia *antigens in stool samples. These lateral flow immunoassays can be accomplished within 10 minutes with a sensitivity more than 97% and specificity of 100% [16].

In Saudi Arabia, few studies have used these immunoassay-based tests for detection of *Giardia *and *Cryptosporidium* in human faecal specimens [17, 18]. In contrary, the traditional microscopic-based techniques, have been widely adopted, in many studies [19–22]. In this study, it was aimed to introduce and subsequently evaluate an immunoassay-based algorithm for screening and identification of *Giardia* and *Cryptosporidium* in human stool samples from Taif, Saudi Arabia. This algorithm is based on three commercial coproantigen kits, namely, TechLab, Inc., *Giardia*/*Cryptosporidium* Chek ELISA kit for *Giardia *and/or *Cryptosporidium* screening in a large number of samples [23], RIDA Quick *Giardia, *and RIDA Quick *Cryptosporidium* cassettes (R-Biopharm) to identify the ELISA-positive samples.

## 2. Material and Methods

### 2.1. Samples Collection and Storage

One hundred sixty diarrhoeal and nondiarrhoeal fresh stool samples were used for preparation of *Giardia*/*Cryptosporidium* positive and negative control samples. Samples were collected from those submitted to the Microbiology Laboratory at King Faisal Public Hospital in Taif, Saudi Arabia, for routine parasitological examination. Additionally, 250 faecal specimens were randomly collected between January and August 2013 for further evaluation and validation of the combined immunoassay-based algorithm. Faecal samples, without any preservatives, were properly labelled and transmitted to the Medical Laboratory at College of Applied Medical sciences, Taif University, within 2-3 hours of collection. Upon arrival to the laboratory, samples were stored at 4°C for microscopic and immunoassays testing. An aliquot of each specimen was prepared, marked, and stored at –20°C for PCR examination.

### 2.2. Control Clinical Samples Preparation

The aforementioned 160 samples were subjected to a combined gold standard test comprising microscopic as well as PCR testing for *Giardia *and *Cryptosporidium*. According to this gold standard test's results, positive and negative samples were classified into control groups.

### 2.3. Protozoan Coproscopic Diagnosis

Unpreserved faecal specimens without prior concentration procedure were subjected to microscopic examination within 2-3 hours after collection. Wet mount preparations, one with saline and the other with iodine, were prepared and each coverslip area was scanned for *Giardia *trophozoite or cyst stages [7]. A third, moderately thick, wet mount smear was prepared from each specimen and subjected to modified Ziehl-Neelsen staining procedure for *Cryptosporidium* oocysts identification [6]. The staining procedure was carried out on batch of samples collected at the same day.

### 2.4. Protozoan Copro-DNA Diagnosis

Aliquots, stored at −20°C, were subjected to DNA extraction within one to two weeks after arrival to the laboratory using QIAmp Stool Mini Kit (Qiagen) following the manufacturer's protocol. DNA extracts were subsequently subjected to amplification by two published PCR assays. PCR assays were conducted following the protocol of Xiao et al. for *Cryptosporidium* and the protocol of Hopkins et al. for *Giardia *[24, 25]. Primers were synthesized by the VHBio (Gateshead, UK). On arrival, primers were dissolved in dH_2_O for stock preparation (100 pmol/*μ*L) and stored at −20°C until use. Reactions were carried out in Techne TC-4000 thermal cycler. GoTaq Hot Start Polymerase (Promega) and other PCR reagents were used in amplification reactions with final concentrations closely similar to the published protocols.

### 2.5. Protozoan Coproantigens Diagnosis

Unpreserved specimens, stored at 4°C, were subjected to examination by the three coproantigen detection kits, under evaluation. Samples were initially screened for *Giardia *and/or *Cryptosporidium* coproantigens using TechLab, Inc., *Giardia*/*Cryptosporidium* Chek ELISA kit. Subsequently, the ELISA-positive specimens were tested for the presence of *Giardia *coproantigen by the RIDA Quick *Giardia *and for *Cryptosporidium* oocysts surface antigen by RIDA Quick *Cryptosporidium* kits. All immunoassays were performed according to the corresponding manufacturers' directions. The rapid tests' results were interpreted visually by the naked eye, while the ELISA test's results were analysed on a multiwell scanning spectrophotometer (ELISA reader) with ≥0.150 being the cutoff for the positive sample at an optical density (OD) of 450 nm.

### 2.6. Data Storage and Statistical Analysis

Results obtained from examination of the clinical stool samples were stored and analysed through Microsoft Excel TM 2007. The diagnostic sensitivity, specificity, positive predictive value, and negative predictive value of various diagnostic assays were determined by standard formulae [26].

## 3. Results

### 3.1. Selection Criteria for Positive and Negative Control Faecal Samples

**Figure 1:**
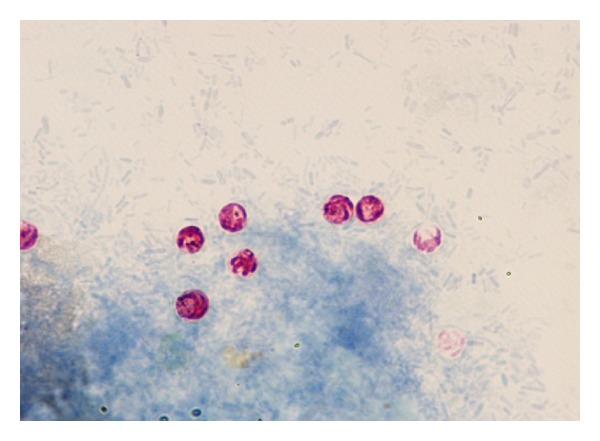
Representative bright field microscopic pictures for the *Cryptosporidium* oocysts stained with modified Ziehl-Neelsen dye with ×200 magnification.

Based on the combined gold standard test-results on the preceding 160 stool samples, three positive control groups were assigned. Samples were diagnosed as positives by microscopy (see Figure 1) and the two PCR assays were selected as a positive control group (group-1).

**Figure 2:**
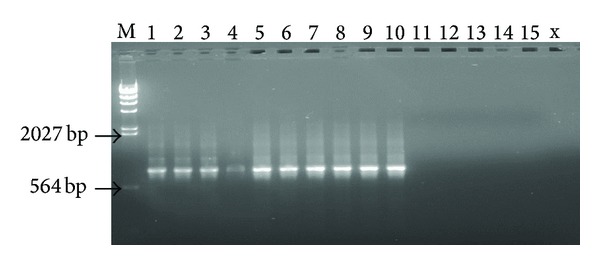
Representative ethidium bromide-stained 1% agarose gel pictures showing amplification products (*≈*825 bp) of *Cryptosporidium* spp., reference nested PCR. Lane 1 to 10, *Cryptosporidium*-positive DNA samples; Lane 11 to 15, *Cryptosporidium*-negative DNA samples; M, *λ*-*Hin*dIII DNA molecular marker.

This group included 25 *Cryptosporidium- * and 30 *Giardia-*positive stool samples. Samples diagnosed as negative by microscopy but positive by the PCR tests (see Figure 2) were selected as a positive control group (group-2). 15 *Cryptosporidium- * and 20 *Giardia-*positive stool samples were included in this group. Finally, stool samples (*n* = 70) diagnosed as *Cryptosporidium- * and *Giardia-*negative by both microscopy and PCR tests were selected as a negative control group (group-3).

### 3.2. The Diagnostic Performance of TechLab ELISA Kit

The screening kit successfully detected the target antigens of all *Cryptosporidium- * and *Giardia-*positive control faecal samples apart from two (see Table 1). These two samples belonged to group-2 and both samples were *Cryptosporidium*-positive by the reference nested PCR. Equally important, the kit showed positive reaction in three samples from group-3, the negative control group (*n* = 70). Interestingly, these three false-positive samples gave no reactions when individually tested by the two subsequent discriminatory rapid kits. Based on these results, the TechLab ELISA test exhibited different diagnostic performance rates for *Cryptosporidium* and *Giardia *as shown in Table 2.

### 3.3. The Diagnostic Performance of RIDA Quick *Giardia*

The kit successfully identified the target antigens of all *Giardia-*positive control faecal samples apart from one (i.e., 49/50). Importantly, this sample was *Giardia *PCR positive but negative by the microscope. No coproantigen for *Giardia* was detected for all negative control samples (*n* = 70). Equally important, no coproantigen for *Giardia *was detected when *Cryptosporidium*-positive control samples (*n* = 40) were examined by the kit. As seen in Table 3, the RIDA Quick *Giardia *kit showed SE, SP, PPV, and NPV of 97.5% (95% CI: from 0.86 to 0.99), 100% (95% CI: from 0.9 to 1.0), 100% (95% CI: from 0.9 to 1.0), and 98.5% (95% CI: from 0.9 to 1. 0), respectively.

### 3.4. The Diagnostic Performance of RIDA Quick *Cryptosporidium* Test

The kit successfully identified the target antigens of all *Cryptosporidium*-positive control faecal samples apart from two (i.e., 38/40). Interestingly, these two samples were those diagnosed as false negatives by the ELISA screening kit. No coproantigen for *Cryptosporidium* was detected for all negative control samples (*n* = 70). Equally important, no coproantigen for *Cryptosporidium* was detected when *Giardia-*positive control samples (*n* = 50) were examined by the kit. As shown in Table 3, the RIDA Quick *Cryptosporidium* kit showed SE, SP, PPV, and NPV of 95% (95% CI: from 0.8 to 0.9), 100% (95% CI: from 0.9 to 1.0), 100% (95% CI: from 0.9 to 1.0), and 97% (95% CI: from 0.9 to 1. 0), respectively.

### 3.5. Validation of the Algorithm by Random Samples

The screening ELISA kit detected *Giardia *and/or *Cryptosporidium* coproantigens in 76 samples. By the second discriminatory kits, *Cryptosporidium* was identified in 24 samples (i.e., 9.6%) and *Giardia *was identified in 42 samples (i.e., 16.8%). Neither *Cryptosporidium* nor *Giardia *coproantigen was identified in the remaining ten samples (4%). Neither *Cryptosporidium* nor *Giardia *copro-DNA was detected when these samples were subjected to diagnosis by the two reference PCR tests. By reviewing the hospital laboratory records regarding these 250 randomly collected samples, *Cryptosporidium* oocysts were identified in 13 samples by microscopic examination of mZN stained wet mounts prepared from fresh unconcentrated specimens. *Giardia *trophozoite and/or cyst stages were detected in 33 samples using microscopic examination of iodine-stained wet mounts prepared directly from fresh faecal specimens. On the other hand, the reference PCR assays accurately detected *Cryptosporidium* DNA in 26 faecal samples and *Giardia *DNA in 42 samples (Table 4). Taking PCR tests' results as a gold standard, the combined immunoassay-based algorithm demonstrated SE, SP, PPV, and NPV of 97.06% (66 of 68; 95% CI: from 0.89 to 0.99), 100.00% (66 of 68; 95% CI: from 0.97 to 1.0), 100.00% (66 of 66; 95% CI: from 0.94 to 1.0), and 98.91% (182 of 184; 95% CI: from 0.96 to 0.99), respectively.

## 4. Discussion

To the best of my knowledge, none of the three coproantigen detection kits has been previously evaluated in Saudi Arabia. In this study, an immunoassay-based algorithm, intended to be used as screening test of *Cryptosporidium*/*Giardia *in stool samples, was appropriately evaluated and shown to meet the defined performance targets. It has to be clear that the differences of a test performance among studies are attributed, to a large extent, to the study methodology followed and population targeted. The prevalence of infection, which differs substantially among populations, affects the predictive value of any diagnostic test. Equally important, the gold standard test used to estimate the diagnostic performance of a diagnostic test varies among studies. A gold standard test with inadequate performance can overestimate false-positive and/or false-negative results of a diagnostic test, under evaluation.

To address the performance characteristics of the algorithm, an assembled reference standard test comprising PCR and microscopy was assembled. Samples were subjected to the combined gold standard test and the results were interpreted as follows. First, stool sample that tested as positive with microscopy was assumed to contain high parasite loads. Second, samples that were negative by microscopy but diagnosed as positive by a reference PCR test were likely to have low to moderate parasite loads. This assumption was taken relying on the previously reported sensitivities of the two diagnostic methods [9, 11, 13, 14, 27]. The TechLab ELISA kit, under evaluation, offered sensitivity towards *Cryptosporidium* of 95%. The kit failed to identify two *Cryptosporidium*-positive specimens. These two samples were microscopically negative but positive with the nested PCR. Interestingly, the optical density readings at 450 nm for the false-negative results were very close to the manufacturer-defined assay cutoff value (0.15). The *Cryptosporidium* oocysts surface antigens being present in these two specimens below the detection limit of the assay may be an explanation to this reduced sensitivity, especially when we know that *Cryptosporidium* oocysts could not be seen microscopically in the mZN stained smears. Chalmers and her colleagues reported sensitivity of 93.4% of the same kit towards *Cryptosporidium* control samples from United Kingdom [28]. In the same study, Remel ProSpect *Giardia*/*Cryptosporidium* and IVD Research *Giardia*/*Cryptosporidium* ELISA kits offered sensitivities of 91.4% and 92.8%, respectively. In many previous studies the kit proved to be 100% specific; however, in this study, it exhibited a slightly lower specificity of 95.71% [28, 29]. This reduced specificity of the TechLab ELISA kit could not be explained. Although the final results of the algorithm were not affected as the samples were ultimately reported as negatives, these false-positive samples required unnecessary retesting.

On the other hand, the RIDA Quick *Giardia *rapid test demonstrated sensitivity and specificity of 97.5% and 100%, a performance which is much better than it has achieved in previous study. Authors at the Institute of Tropical Medicine in Berlin, Germany, in 2006, have reported sensitivity and specificity figures of 80% and 99.4%, respectively [30]. Similarly, RIDA Quick *Cryptosporidium* lateral flow test, used in the study, showed higher performance than it offered previously. Equally important, the RIDA Quick *Cryptosporidium* did not cross-react with stool samples containing *Giardia *and RIDA Quick *Giardia *kit did not cross-react with *Cryptosporidium*-positive stool samples. Previous studies have reported false-positive *Cryptosporidium* infections with other rapid tests [31, 32]. Others have demonstrated higher specificity of 99.0%–100.0, closely similar to the rapid kits, evaluated in this study [33, 34].

Further evaluation of the algorithm was carried with a fairly large number of random stool samples with blinded microscopic test results as generated by routine testing procedures at the Clinical Microbiology Laboratory. Based on tests' results on randomly collected samples, the immunoassay-based algorithm performance was comparable to two PCR tests' results and much better than the two traditional staining methods used for *Giardia*/*Cryptosporidium* diagnosis. This performance rates achieved by the algorithm were higher than those reported for the individual kits, under evaluation, [28, 30] and others immunoassay-based kits [9, 11, 13, 14, 27]. *Cryptosporidium* coproantigen was confirmed in 24 samples (9.6%) and *Giardia *coproantigen was confirmed in 42 (16.8%). These prevalence rates of infections do not reflect the true prevalence of these two protozoa in Taif city because samples were collected within a short period. Ten samples were positive by the ELISA kit but were negative for *Giardia *or *Cryptosporidium* coproantigens by the two rapid tests. None of these samples was positive for *Giardia *or *Cryptosporidium* by the reference PCR assays and microscopy. It is clear that the high specificities offered by the two rapid tests counterbalanced the unwanted false-positive results of the ELISA kit and, as a result, the overall algorithm test results were not affected.

## 5. Conclusion

The immunoassay-based algorithm performed well with the collected stool samples. It was proved to be a very useful screening tool for the target specific protozoan antigens in stool specimen. The algorithm showed diagnostic performance higher than those achieved by the classical staining methods. Its results were comparable to those given by the reference PCR assays used in the study. Bearing in mind the large number of samples that can be screened, the ease of use, the less hands-on time, and its amenability for future semiautomation, the introduced algorithm has a real potential to be adopted in diagnostic laboratories, especially those having a large number of specimens likely to be tested.

## Figures and Tables

**Table 1:** The ELISA kit's results compared to the assembled gold standard test's results.

ELISA kit results	The combined gold standard test results^a^		
	Positive	Negative	Total
Positive	88	3^c^	91
Negative	2^b^	67	69

Total	90	70	160

^a^The combined gold standard test comprising PCR assays and microscopy.

^b^These two samples with false-negative test results were diagnosed as negative by microscopy but *Cryptosporidium*-positive by the reference PCR assay (group-2).

^c^These three samples with false-positive test results were PCR negative for *Giardia *and *Cryptosporidium* and gave no reactions when individually tested by the two subsequent rapid tests.

**Table 2:** The diagnostic performance of the ELISA kit for *Cryptosporidium* and *Giardia. *

Genus	The diagnostic performance parameters							
	Sensitivity		Specificity		PPV		NPV	
	SE%	95% CI	SP%	95% CI	PPV%	95% CI	NPV%	95% CI
*Giardia *	100%	0.9-1.0	95.71%	0.8-0.9	94.34%	0.8-0.9	100%	0.9-1.0
*Cryptosporidium *	95%	0.8-0.9	95.71%	0.8-0.9	92.68%	0.8-0.9	97%	0.8-0.9

**Table 3:** The diagnostic performance of the two rapid tests.

RIDA Quick	The diagnostic performance parameters							
	Sensitivity		Specificity		PPV		NPV	
	SE%	95% CI	SP%	95% CI	PPV%	95% CI	NPV%	95% CI
*Giardia *	97.5%	0.8-0.9	100%	0.9-1.0	100%	0.9-1.0	98.5%	0.9-1.0
*Cryptosporidium *	95%	0.8-0.9	100%	0.9-1.0	100%	0.9-1.0	97%	0.9-1.0

**Table 4:** Diagnostic tests' results on 250 random stool samples.

Test result	Microscopy	The algorithm^a^	The reference PCRs^b^
*Cryptosporidium*-positive	13	24	26
*Giardia-*positive	33	42	42
*Crypto^c^*/*Giardia*-negative	204	184	182
Total	**250**	**250**	**250**

^a^The algorithm picked 20 positive samples more than the staining methods adopted at the Microbiology Laboratory at King Faisal Hospital. These samples were also positive for protozoan DNA by the matching reference PCR assay. ^b^Two *Cryptosporidium* DNA positive samples were diagnosed by PCR and missed by the algorithm. ^c^ *Crypto* stands for *Cryptosporidium*.
